# Rapid Development and Characterization of Chromosome Specific Translocation Line of *Thinopyrum elongatum* with Improved Dough Strength

**DOI:** 10.3389/fpls.2017.01593

**Published:** 2017-09-14

**Authors:** Aman Kumar, Monika Garg, Navneet Kaur, Venkatesh Chunduri, Saloni Sharma, Swati Misser, Ashish Kumar, Hisashi Tsujimoto, Quan-Wen Dou, Raj K. Gupta

**Affiliations:** ^1^National Agri-Food Biotechnology Institute Mohali, India; ^2^United Graduate School of Agriculture, Tottori University Tottori, Japan; ^3^Northwest Institute of Plateau Biology (CAS) Qinghai, China; ^4^Indian Institute of Wheat and Barley Research, Indian Council of Agricultural Research Karnal, India

**Keywords:** wheat, translocation line, HMW glutenin, wide hybridization, bread making quality, *Thinopyrum elongatum*, genomic *in situ* hybridization (GISH), marker assisted selection (MAS)

## Abstract

The protein content and its type are principal factors affecting wheat (*Triticum aestivum*) end product quality. Among the wheat proteins, glutenin proteins, especially, high molecular weight glutenin subunits (HMW-GS) are major determinants of processing quality. Wheat and its primary gene pool have limited variation in terms of HMW-GS alleles. Wild relatives of wheat are an important source of genetic variation. For improvement of wheat processing quality its wild relative *Thinopyrum elongatum* with significant potential was utilized. An attempt was made to replace *Th. elongatum* chromosome long arm (1EL) carrying HMW-GS genes related to high dough strength with chromosome 1AL of wheat with least or negative effect on dough strength while retaining the chromosomes 1DL and 1BL with a positive effect on bread making quality. To create chromosome specific translocation line [1EL(1AS)], double monosomic of chromosomes 1E and 1A were created and further crossed with different cultivars and homoeologous pairing suppressor mutant line *Ph*^*I*^. The primary selection was based upon glutenin and gliadin protein profiles, followed by sequential genomic *in situ* hybridization (GISH) and fluorescent *in situ* hybridization (FISH). These steps significantly reduced time, efforts, and economic cost in the generation of translocation line. In order to assess the effect of translocation on wheat quality, background recovery was carried out by backcrossing with recurrent parent for several generations and then selfing while selecting in each generation. Good recovery of parent background indicated the development of almost near isogenic line (NIL). Morphologically also translocation line was similar to recipient cultivar N61 that was further confirmed by seed storage protein profiles, RP-HPLC and scanning electron microscopy. The processing quality characteristics of translocation line (BC_4_F_6_) indicated significant improvement in the gluten performance index (GPI), dough mixing properties, dough strength, and extensibility. Our work aims to address the challenge of limited genetic diversity especially at chromosome 1A HMW-GS locus. We report successful development of chromosome 1A specific translocation line of *Th. elongatum* in wheat with improved dough strength.

## Introduction

Wheat is one of the major cereal food crops with worldwide consumption. More than 700 million metric tons of wheat have been produced globally each year since 2013 which means it accounts for more than 30% of total cereal production and provides almost 20% of total calorific input to the world (USDA/ARS, 2017). Wheat (2n = 6X = 42) contains three closely related genomes: A, B, and D that are derived from three different diploid species (Feldman and Levy, 2015). Wheat flour protein combination distinguishes it from other cereals as it confers properties of viscoelasticity and extensibility to the dough (Wang et al., 2013). Gliadins and glutenins are two main components of seed storage proteins that build the gluten polymer and determine its properties (Shewry, 2009). Gliadins are monomeric proteins that impart extensibility to the dough. These can be categorized into α/β, γ, and ω gliadins. Glutenins are polymeric proteins that impart dough its viscoelasticity. Glutenins can be classified into high molecular weight glutenin subunits (HMW-GS) and low molecular weight glutenin subunits (LMW-GS). HMW-GS confer dough strength while the LMW-GS are responsible for dough extensibility (Wang et al., 2013). The loci encoding HMW-GS and LMW-GS are located on the long and short arms, respectively, of homoeologous group 1 chromosomes: 1A, 1B, and 1D (Sreeramulu and Singh, 1997). Each locus produces two subunits of different sizes, larger x-type and smaller y-type subunits. The variation in different HMW-GS is due to variation in size and number of cysteine residues (Shewry and Tatham, 1997). Subunits 1B_x_, 1D_x_, and 1D_y_ are expressed in all wheat cultivars while 1B_y_ and 1A_x_ subunits are expressed in some wheat cultivars. Different alleles of these subunits have different impact on end product quality and have accordingly been given different quality scores (Payne and Lawrence, 1983). 1D_x_5+1D_y_10 subunits from D genome and 1B_x_17+1B_y_18, 1B_x_13+1B_y_16, and 1B_x_14+1B_y_15 from B genome are associated positively with bread making quality as compared to other corresponding alleles (Mills et al., 2000; Branlard et al., 2001; Tohver, 2007). The gene coding 1A_y_ subunit generally remains silent in most of the wheat cultivars (Rasheed et al., 2014). Chromosome 1A (*Glu-A1*) is reported to be the least contributor (Zhang et al., 2016) and may affect processing quality negatively in some backgrounds (Garg et al., 2007). The *Glu-A1* loci thus can be targeted for introgression of new alleles from different genetic resources. Wild species of wheat have rich HMW-GS variations (Wang et al., 2013) and may provide potential quality gene sources for this purpose. Being a polyploid, wheat has a highly buffered genome that is tolerant to alien introgressions. Wheat has been targeted for improvement in yield, disease resistance, abiotic stress tolerance, nutrition, and processing quality (Wulff and Moscou, 2014). To accomplish sufficient yield and resistance to diseases, many economically important genes have been transferred to wheat through chromosome translocations from many different relative genera (Feldman and Levy, 2015; Ma et al., 2015) such as, resistance genes Pm53, Yr40, and Yr42 from *Aegilops* spp. (Kuraparthy et al., 2009; Marais et al., 2009; Petersen et al., 2015), Pm21 from *Haynaldia villosa* (Chen et al., 1995), Pm40 and Yr50 from *Thinopyrum* (Luo et al., 2009; Liu et al., 2013) and YrSn0096 from *Leymus mollis* (Bao et al., 2012). *Secale cereal* (rye) specific translocations have been used to transfer both yield and resistance genes (Yr9, Pm8, Lr26, Sr31) into wheat (Mago et al., 2005; Ren et al., 2009, 2017). The rye-wheat 1RS.1BL translocation was used worldwide in wheat breeding programs and is well distributed as cultivars across the world (Rabinovich, 1998). It has substantially contributed to yield enhancement, productivity increase, and has affected agricultural economy (Rabinovich, 1998). Despite their wide use in conferring resistance traits to wheat cultivars not too many translocation lines have been developed for quality traits such as, dough strength and extensibility. T1BL.1RS is known to have a negative impact on bread making quality (Kumlay et al., 2003).

*Thinopyrum elongatum* (syn. *Agropyron elongatum*) also known as tall wheat grass belongs to family Poaceae. It has a diploid E genome (2n = 2X = 14) that can be utilized for increasing the genetic diversity of HMW-GS genes (Chen et al., 2013; Royal Botanic Gardens Kew, 2017). *Th. elongatum* has been used to transfer genes for salinity, drought, and disease resistance to wheat in many breeding programs (Dewey, 1960, 1962; Sharma and Gill, 1983; Colmer et al., 2006). Yield related genes from *Thinopyrum* have also been transferred in form 7DL.7Ag translocation (Monneveux et al., 2003). Wheat-*Th. elongatum* translocations have been used for conferring resistance to leaf rust and stem rust (Friebe et al., 1994). The disomic addition line of chromosome 1E (DAL1E) of *Th. elongatum* in Chinese spring (CS) wheat cultivar background have been reported to possess superior bread making quality (Garg et al., 2009a). The presence of whole alien chromosomes may result in reduced yield and the line may be genetically unstable (Zamir, 2001). Translocation lines are appropriate alternative to DALs/DSLs for introgression of wild alleles. Our previous work on *Th. elongatum* and *Aegilops geniculata* substitution lines have indicated that positive processing quality potential of wild species can be best utilized if translocation is carried out by replacing chromosome 1A specific HMW-GS rather than those coded by 1B and 1D (Garg et al., 2009b, 2016). The current study was aimed at replacing *Glu-A1* loci with better quality genes from wheat related species. We developed chromosome 1A specific translocation line of *Th. elongatum* in wheat background. The effects of this translocation on processing quality were studied.

## Materials and methods

### Plant materials

The plant materials utilized in this study included (1) disomic addition line of *Th. elongatum* chromosome 1E and disomic substitution line of *Th. elongatum* chromosome 1E with chromosome 1D of wheat, both in the background of Chinese Spring (CS) {DSL-1E(1D)}, (2) genetic stocks nulli for chromosome 1A and tetra for chromosome 1D (N1AT1D), N1AT1B, N1BT1A, N1BT1D, and N1DT1B in the background of CS, and (3) four cultivars: AcDomain-hard Canadian, Norin 61 (N61)-soft Japanese, PBW343-hard Indian, Cham 6-hard Syrian.

### Generation of chromosome specific translocation line

Search for chromosome specific protein markers was accomplished from electrophoretic patterns of glutenin and gliadin proteins of *Th. elongatum* addition and substitution lines, and nulli tetra genetic socks of CS. For creation of translocation line, the DSL-1E(1D) was reciprocally crossed with N1AT1D to create double monosomics for chromosome 1E and 1A. The F_1_ generation was crossed/backcrossed with cultivars N61, CHAM6, PBW343, AcDomain, and with *Ph*^*I*^ mutant line (promote homoeologous pairing and recombination). Selection was based upon presence of HMW-GS and absence gliadins from *Th. elongatum* and absence of 1AS coded gliadins from wheat. Confirmation of translocation was carried out by GISH analysis. Plants with high vigor were selected and additional cycles of crossing/backcrossing and selfing were carried out.

### Protein characterization

Seed storage proteins were sequentially extracted according to Smith and Payne (1984) with modifications. Initially gliadin extraction was accomplished with 1.5 M DMF (dimethyl formamide), followed by glutenin extraction with 50% Isopropanol, 50 mM TrisHCl (pH 7.5), 2% DTT. Separation of glutenins was carried out on 10% polyacrylamide gel. Gliadins were separated on 15% polyacrylamide gels.

### Reversed phase high performance liquid chromatography (RP-HPLC)

RP-HPLC of separated gliadin and glutenin proteins was carried out according to Mejias et al. (2014) with some modifications. The separation of proteins was carried out using C8 reversed phase analytical column Zorbax 300SB-C8 (Agilent Technologies) with 5 μm particle size and 300 Å particle diameter, 250 mm length, 4.6 mm internal diameter of Agilent 1260 Infinity Quaternary liquid chromatography system. The detector was diode array UV-vis detector and absorbance was detected at 210 nm. Injection volume was 20 μl. Linear gradient was set up using solvents A (0.1% TFA in MQ) and B (0.1% TFA in Acetonitrile) and column temperature was set up at 60°C. Glutenins were separated at a linear gradient 25–44% B for 120 min, 44–50% B for 5 min, and finally 50–65% B for 5 min. Gliadins were separated from 20 to 65% B for 120 min and then 65 to 75% B for 20 min.

### Background screening

Background screening of the translocation line was done by simple sequence repeat (SSR) markers. Total 536 deletion bin based primers (Sourdille et al., 2004; Carollo et al., 2005) were synthesized and used for background screening of BC_4_F_3_ plants (Supplementary Datasheet). N61, CS and 1E(1D) were used as controls. The genomic DNA of the selected samples was isolated using CTAB (Cetyl trimethyl ammonium bromide) method (Doyle and Doyle, 1987). The amplification was carried out in 20 μl of reaction mixture containing genomic DNA, PCR Mastermix (Thermo Fisher Scientific), and 0.4 μM primers using a thermal cycler (Eppendorf). Thermal cycling program involved an initial denaturation at 95°C for 5 min, followed by 32 cycles of denaturation at 95°C for 60 s, annealing at 5°C below the Tm of respective primers for 30 s, primer extension at 72°C for 30 s, followed by a final extension at 72°C for 10 min. The amplified PCR products were size fractioned by electrophoresis on 2.5% agarose gel and/or 10% polyacrylamide gel and visualized by staining with ethidium bromide (0.5 μg/ml) in a gel documentation system (Biospectrum UVP).

### Scanning electron microscopy (SEM)

Developing seeds of N61 and translocation line were collected at four different stages (7DAA, 18DAA, 25DAA, and 28DAA). For SEM, seeds were embedded in the optimal cutting temperature (OCT) media (Leica biosystems) and kept for freezing for half an hour. Then the cryosectioning of the embedded seeds was carried out at −20°C in a cryomicrotome (Leica CM1950 cryostat). For carrying out scanning electron microscopy, the cryosections of 40 μm thickness were collected on the adhesive carbon strip and coated with gold particles. After coating, the samples were mounted on the scanning electron microscope and observed. For the mature seeds, instead of performing cryosectioning, the seeds were cracked under liquid nitrogen. The cracked seeds were then adhered onto the adhesive carbon strip. The rest of the procedure was same as in the case of the developing seeds.

### Cytological analysis

The Acetocarmine squash method (Tsuchiya and Nakamura, 1979) was utilized for the preparation of mitotic chromosome spreads. *Th. elongatum* genomic DNA was isolated by CTAB method (Doyle and Doyle, 1987). Genomic *in situ* hybridization (GISH) and fluorescent *in situ* hybridization (FISH) were carried out for screening and confirmation of translocation line. Total genomic DNA of *Th. elongatum* was labeled with fluorescein-12-dUTP (Roche) or tetramethyl-rhodamine-5-dUTP (Roche) by random primer labeling method and was used as a probe to identify the translocation (GISH). pAS1 and AAG sequences labeled with cyanine (Cy3) and 6-fluorescein amidite (6-FAM). respectively, were used as probes for FISH to identify specific chromosomes. Chromosomes were counterstained with 4′,6-diamidino-2-phenylindole (DAPI) (Roche) and observed under the fluorescence microscope.

### Grain quality analysis

The moisture, protein content, dry gluten, and wet gluten of milled flour were determined according to standard American Association of Cereal Chemists (AACC, 1990) methods. Sodium dodecyl sulfate sedimentation (SDSS) values were measured on a small scale using 1 g of flour by the method of Takata et al. (1999). Dough extensibility tests (Totosaus et al., 2013) were performed on texture analyzer (Stable Microsystems) using Kieffer dough and gluten extensibility rig. The values of peak positive force, stretching distance, area to positive peak and force at the target were calculated for different dough samples. Solvent retention capacity (SRC) tests were performed according to AACC International Method 56-11.02 for deionized water, sucrose solution (50% w/w), sodium carbonate solution (5%w/w), and lactic acid solution (5%w/w). To determine mixing properties of flour a 10 g mixograph (National Mfg. Co., Lincoln, NE, USA) was used. Mixing tests were performed in triplicates using AACC method 54-40A. Results were analyzed using MixSmart software (AEW Consulting, Lincoln, NE, USA).

### Statistical analysis

To determine the level of significance of different parameters, analysis of variance (ANOVA) was performed using the stat view program.

## Results

### Generation of chromosome specific translocation line DTL-1EL(1AS)

For generation of chromosome specific translocation line *Th. elongatum* substitution line DSL-1E(1D) and N1AT1D were utilized. As both are in the genetic background of CS, their glutenin, and gliadin seed storage protein electrophoretic profiles were studied for identification of protein markers (Supplementary Figure 1). Based on glutenin profile *Th. elongatum* specific HMW-GS were identified and further utilized as markers for screening of 1EL chromosome. From gliadin profile *Th. elongatum* specific gliadins were identified and utilized as 1ES specific marker. The HMW-GS profiling of CS indicated that it has a null allele at *Glu-A1* locus. Therefore, LMW glutenin and gliadin profiles were explored for chromosome 1A specific protein subunits by utilizing its nullitetra stocks N1AT1B, N1AT1D, N1BT1A, N1BT1D, and N1DT1B. One, chromosome 1AS specific gliadin was identified and used as a protein marker for screening IAS chromosome (Supplementary Figure 2).

**Figure 1:**
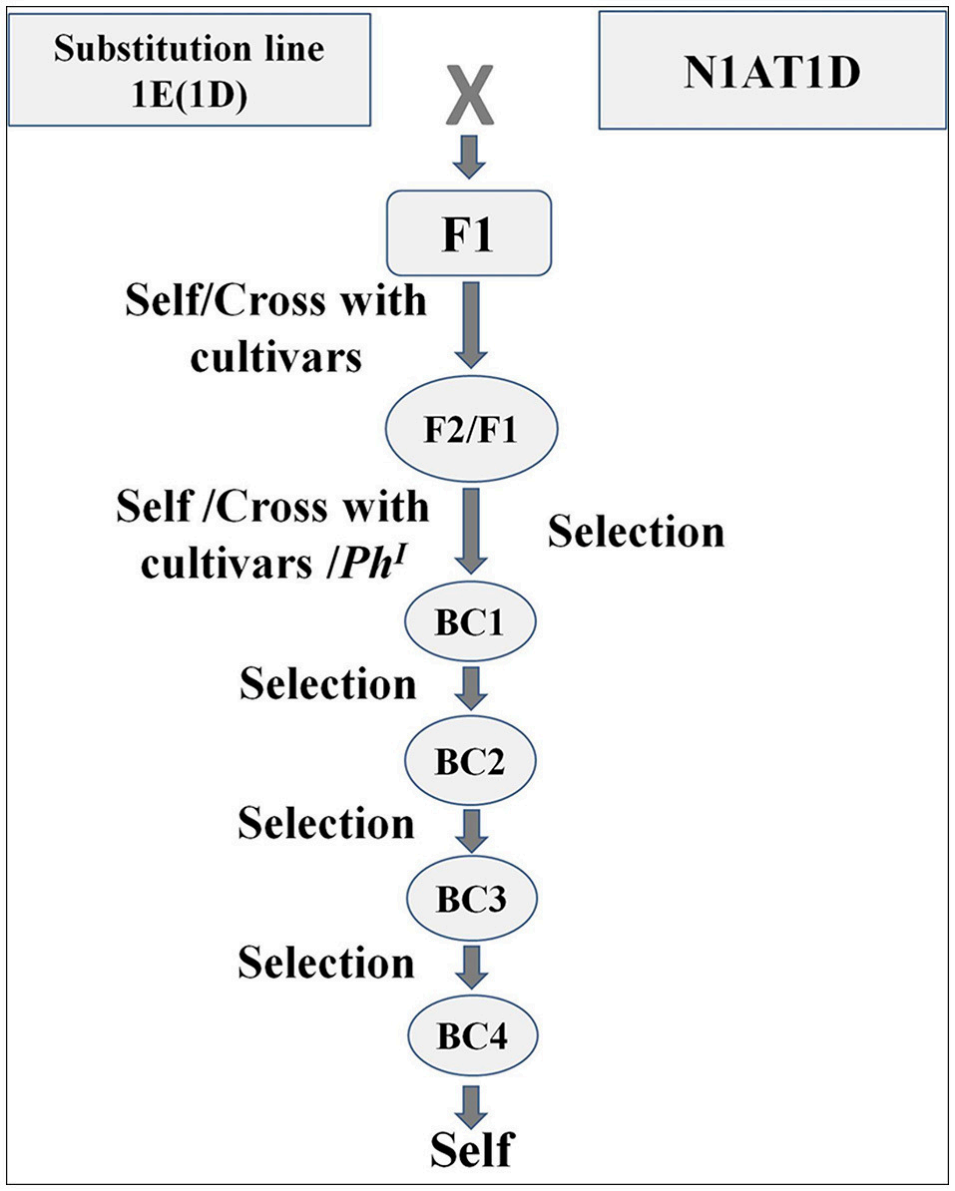
Schematic representation of crossing scheme involved in the generation of chromosome specific translocation line [1EL (1AS)]. Double monosomics were created by carefully selecting the breeding material and screening was carried out by combination of protein markers and genomic *in situ* hybridization. Multiple rounds of backcrossing were carried out to recover the background of recipient cultivar. N1AT1D = Genetic stock nullisomic for chromosome 1A and tetrasomic for chromosome 1D.

For a generation of translocation line the DSL-1E (1D) was reciprocally crossed with N1AT1D (Figure 1). The F_1_ was double monosomic for 1E and 1A and was supposed to produce a translocation line at very low frequency during next meiosis. The F_1_ plants were selfed and crossed with cultivars N61, Cham6, PBW343, AcDomain. The endosperm halves of selfed (F_2_ of N1ATID cross) and F_1_generations from crosses with cultivars were analyzed for HMW-GS and gliadins profile for the screening of translocation line (Supplementary Figure 3). Out of 243 analyzed seeds 108 carried *Th. elongatum* HMW-GS. Expression of CS specific chromosome 1A coded gliadins was not observed in 87 of total analyzed seeds. Total 21 seeds which showed expression of *Th. elongatum* HMW-GS and absence of CS specific chromosome 1A coded gliadins were selected for further study. Embryo halves of these seeds were sown in petri plates, their two roots were collected and the rest of the seedling was sown in the pot to raise plant. Collected roots were fixed and analyzed by GISH with genomic DNA of *Th. elongatum*, AAG and pAS1 sequences. Nineteen seeds including double monosomics and disomic substitution 1E(1A) were selected. Selection included six plants from AcDomain cross, two from Cham 6 cross, two from Norin61 cross, showing monosomic substitution (double monosomics), and nine from self (F_2_) showing disomic substitution. No translocations could be identified at this stage. We could recover two healthy plants with healthy spikes from AcDomain cross, two from self and one from Norin61 cross. As we could not identify any translocation line, to increase the chance of homoeologous recombination, these plants were crossed with *Ph*^*I*^ positive line. These plants were also crossed with AcDomain, N61 depending on the cross. Total 174 BC_1_/F_1_ seeds were screened for HMW-GS and gliadin profiles from the endosperm half while embryo part was used to get roots for GISH/FISH and raise the plants. Out of 76 seeds analyzed from AcDomain (BC_1_), four seeds having HMW-GS of *Th. elongatum* and missing gliadins of *Th. elongatum* were selected and used for further studies (Table 1). Similarly six out of 88 analyzed F_1_ seed from *Ph*^*I*^ positive line cross from the initial AcDomain crosswere selected. We could select only three seeds out of 20 analyzed seeds from N61 cross (BC_1_). These 13 selected seeds were checked by GISH analysis. Out of this one BC_1_ seed from N61 cross and one F_1_seed from *Ph*^*I*^ positive line cross from AcDomain cross were found to be translocation lines with 1EL from *Th. elongatum* and 1AS from wheat. Apart from translocation, we observed broken chromosomes, and missing *Th. elongatum* chromosomes in other 11 seeds. Translocation line development frequency was 1.1% from AcDomain/*Ph*^*I*^ cross and 5% from N61 backcross (Table 1). But screening efficiency by GISH was increased to 16.7% in AcDomain/*Ph*^*I*^ cross and 33.3% in N61 backcross after first screening by protein markers. These selected seeds were grown and further backcrossed with N61 or AcDomain depending on the cross, and selected by HMW-GS profile. AcDomain/*Ph*^*I*^ cross specific translocation line could not be carried forward due to poor plant growth and seed setting. The *Ph*^*I*^ selfed progeny could not produce viable plants and could not be used for screening of translocation line, thus only one translocation from N61 cross was selected. N61 cross specific translocation line was backcrossed four times and selfed. HMW-GS specific marker was used in each generation. Homozygous line was selected in the F3 generation by screening at least 10 seeds from individual F_2_ plants. One healthy F_2_ plant with all seeds showing 1E specific HMW-GS was considered homozygous. Individual F_3_ plants raised from these seeds were again checked for homozygosity and one healthy plant was selected for generation advancement and selfed until F_6_ generation. These BC_4_F_6_ seeds were used for further analysis.

**Table 1:** Frequency of generation of chromosome specific translocation [1EL(1AS)] in different crosses.

**Cross**	**No. of seeds analyzed**	**No. of seeds selected (SDS PAGE)**	**No. of seeds selected (GISH)**	**Percentage of translocated chromosome/screening efficiency**
1E(1D) X N1AT1D X AcDomain X AcDomain	76	4	0	0
1E(1D) X N1AT1D X AcDomain X *Ph^*I*^*	88	6	1	1.14/16.66
1E(1D) X N1AT1D X N61 X N61	20	3	1	5/33.3

*Screening efficiency by GISH was increased after first screening by protein markers*.

### Identification of translocations

#### Electrophoretic profile of storage proteins

**Figure 2:**
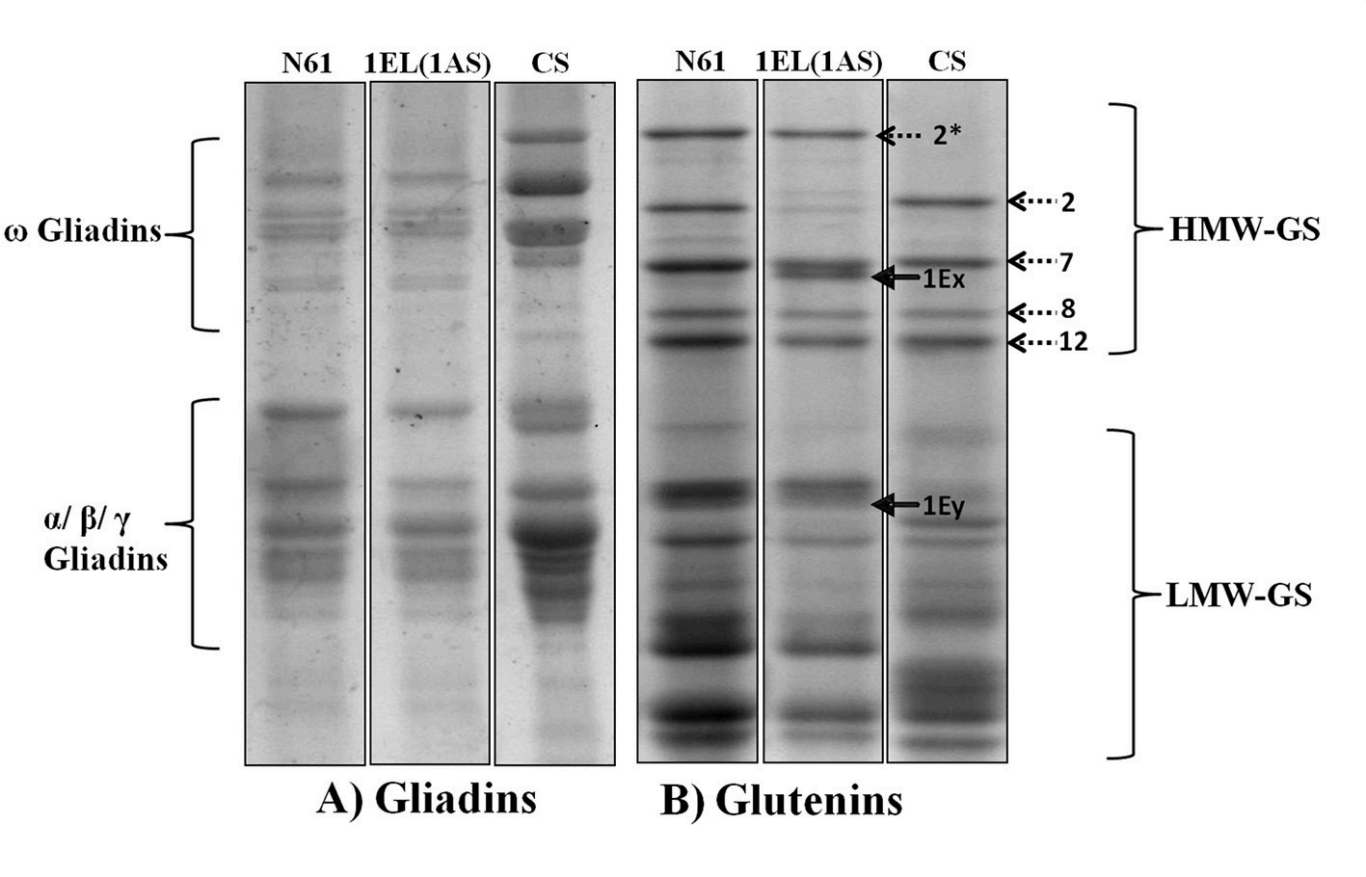
Seed storage protein electrophoretic profile of N61, CS, and DTL-(1EL1AS) **(A)** Gliadins profile; **(B)** Glutenins profile; arrow showing *Th. elongatum* specific protein band (1Ex). Electrophoretic profile indicated replacement of N61 chromosome 1AL coded HMW-GS with those of *Th. elongatum*. No difference was observed in the gliadin protein profiles of N61 and DTL-(1EL1AS).

The seed storage protein profile via SDS-PAGE indicated that DTL-1EL(1AS) had six HMW-GSs, four from N61 (1D_x_2.2, 1D_y_12, 1B_x_7, 1B_y_8), and two from *Th. elongatum* (1E_x_ and 1E_y_; Figure 2). *Th. elongatum* specific 1E_x_ band was located between 1B_x_7 and 1B_y_8. The 1E_y_ band was located in the LMW glutenin region. Expression of 1A_x_2^*^ observed in N61 was not found in DTL-1EL(1AS). No differences were observed in the gliadin pattern of parent cultivar N61 and translocation line 1EL(1AS).

### RP-HPLC of storage proteins

**Figure 3:**
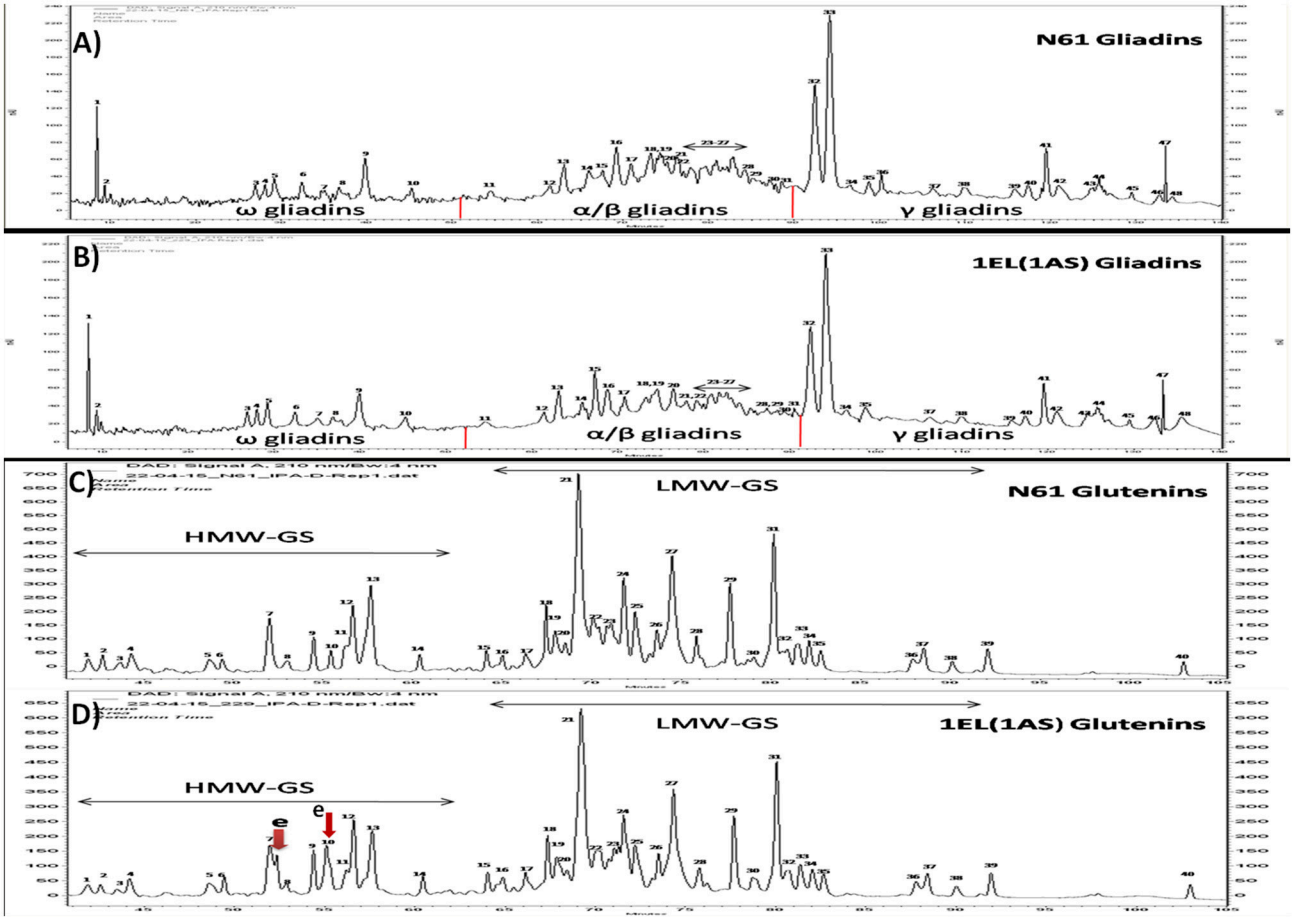
RP-HPLC analysis of gliadins fraction of **(A)** N61, **(B)** DTL-1EL(1AS) and glutenin fractions of **(C)** N61, **(D)** DTL-1EL(1AS). Two additional peaks were observed in the HMW-GS part of the HPLC profile (indicated by red arrows). Gliadin peak patterns of N61 and translocation line were similar.

RP-HPLC of glutenin fraction was done using a linear gradient (Figure 3). The proteins separated between RT-values 30–105 min. Total 40 major peaks were observed in the chromatogram. HMW-GS specific peaks could be detected between RT-values of 30–62 min. In HMW specific region 14 peaks were identified. There was one additional *Th. elongatum* specific peak in the glutenin fraction of translocation line 1EL(1AS) in the HMW GS region between peaks 7 and 8. Second peak was overlapping with peak 10. LMW-GS specific peaks could be detected between RT-values of 62–105 min. A total number of 26 peaks were identified in the LMW region. No differences were observed in LMW-GS specific region of glutenin fraction of translocation line and recipient cultivar N61.

The separation of gliadins fraction occurred between RT-values of 7–140 min. Three regions could be identified in the chromatogram: ω gliadins specific region between RT-values of 7–52 min, α/β gliadins specific region between 53 and 91 min, and γ gliadin specific region between 92 and 140 min. Ten peaks were observed in ω gliadins region while 21 peaks could be identified in α/β gliadins region. Total 17 major peaks were detected in γ gliadins region. No differences were observed in gliadin fractions of both N61 and 1EL(1AS) translocation line.

### Cytological analysis

**Figure 4:**
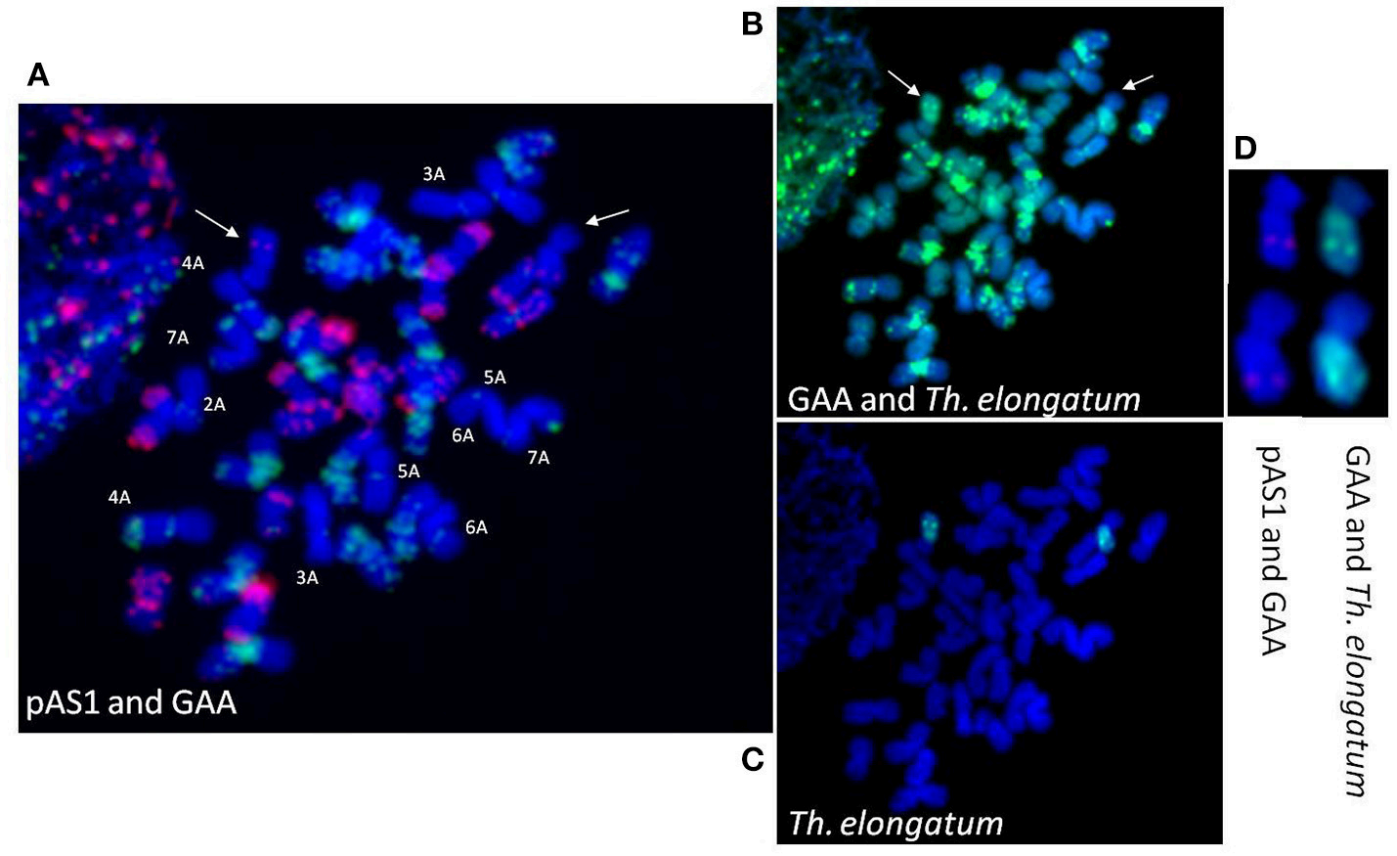
Genomic *in situ* hybridization for detection of *Thinopyrum elongatum* specific translocation in *Triticum aestivum*; **(A)** pAS1 (red) and GAA (green) signals for screening of individual wheat chromosomes; **(B)** GAA (green) and *Th. elongatum* (green marked by arrow) signals for screening of individual wheat chromosomes; **(C)** *Th. elongatum* specific signals (green) for screening of translocation line; **(D)** pAS1 (red), GAA (green), and *Th. elongatum* signals (green) detecting 1EL(1AS). Chromosomes were counterstained with DAPI (blue).

Sequential GISH and FISH analysis was carried out to confirm the translocation. First GISH with *Th. elongatum* genomic DNA was carried out (Figure 4C) and then both *Th. elongatum* genomic DNA and GAA sequences were used (Figure 4B). It was followed by FISH with GAA and pAS1 probes (Figure 4A). GISH analysis using *Th. elongatum* genomic DNA as probe revealed that *Th. elongatum* chromosome was translocated to one of the wheat chromosomes. It showed presence of Robertsonian translocation i.e., a whole arm of wheat chromosome was replaced with that of *Th. elongatum* (Figures 4C,D). The long arm of *Th. elongatum* was transferred to short arm of one of wheat chromosomes. Identity of wheat chromosomes was revealed by pAS1 and GAA FISH probes. These sequences give specific banding signals on individual chromosomes which can be utilized to identify them. All the 14 D-genome chromosomes were identified with pAS1 signals (Figure 4A) indicating that D-genome was not involved in translocation. GAA sequences could identify all the 14 B-genome chromosomes so B-genome involvement was also ruled out. Only 12 A-genome chromosomes (2A to 7A) could be identified from the GAA pattern (Figure 4A). It demonstrated that the long arm of *Th. elongatum* chromosome was attached to short arm of chromosome 1A of wheat.

### Background screening

**Figure 5:**
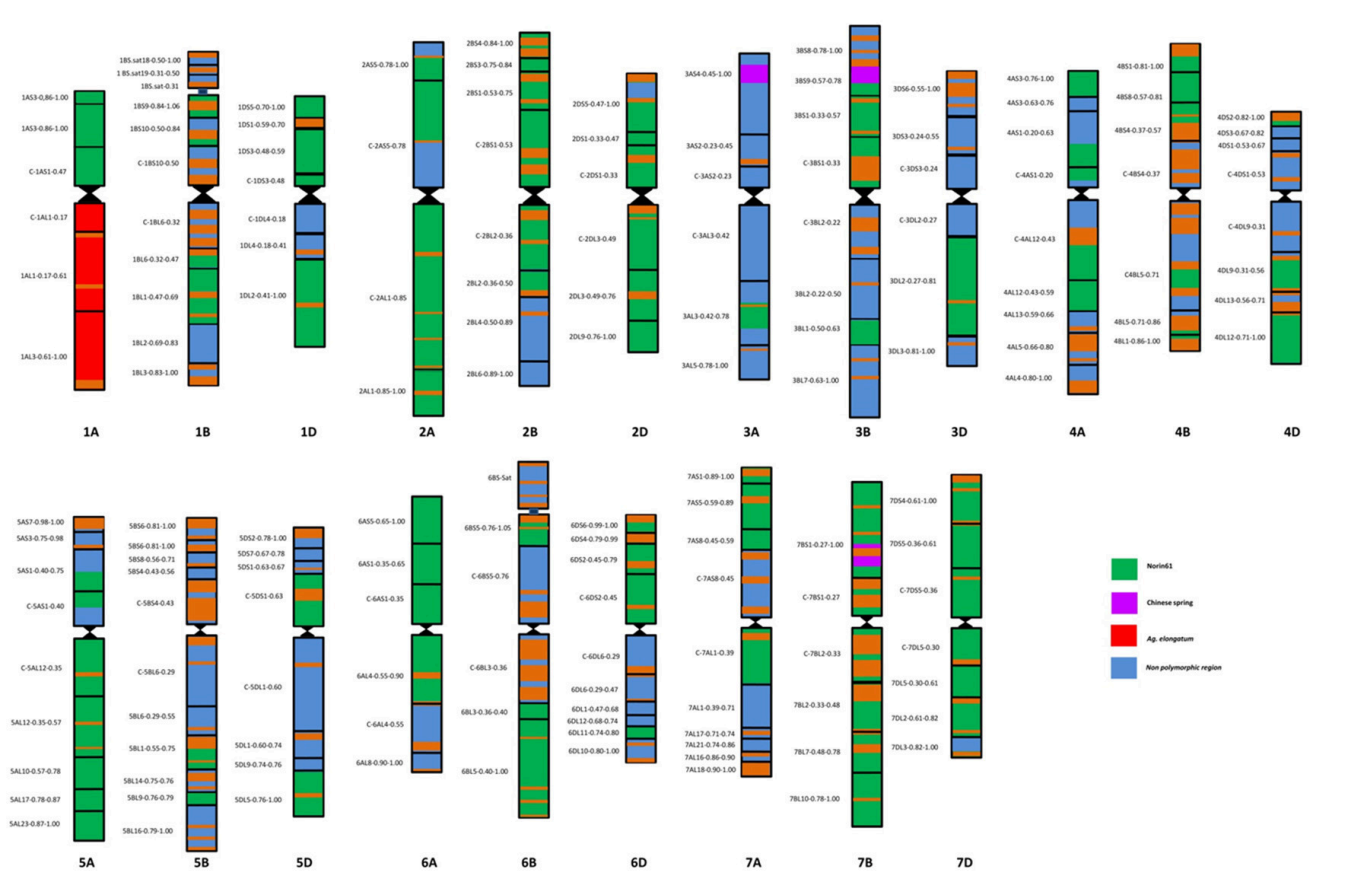
Schematic microsatellite marker based map of DTL-1EL (1AS) translocation lines; map showing N61 (green), CS (purple), and *Th. elongatum* (red) backgrounds in DTL-1EL(1AS). Good background recovery of N61 indicated generation of almost near isogenic line.

**Figure 6:**
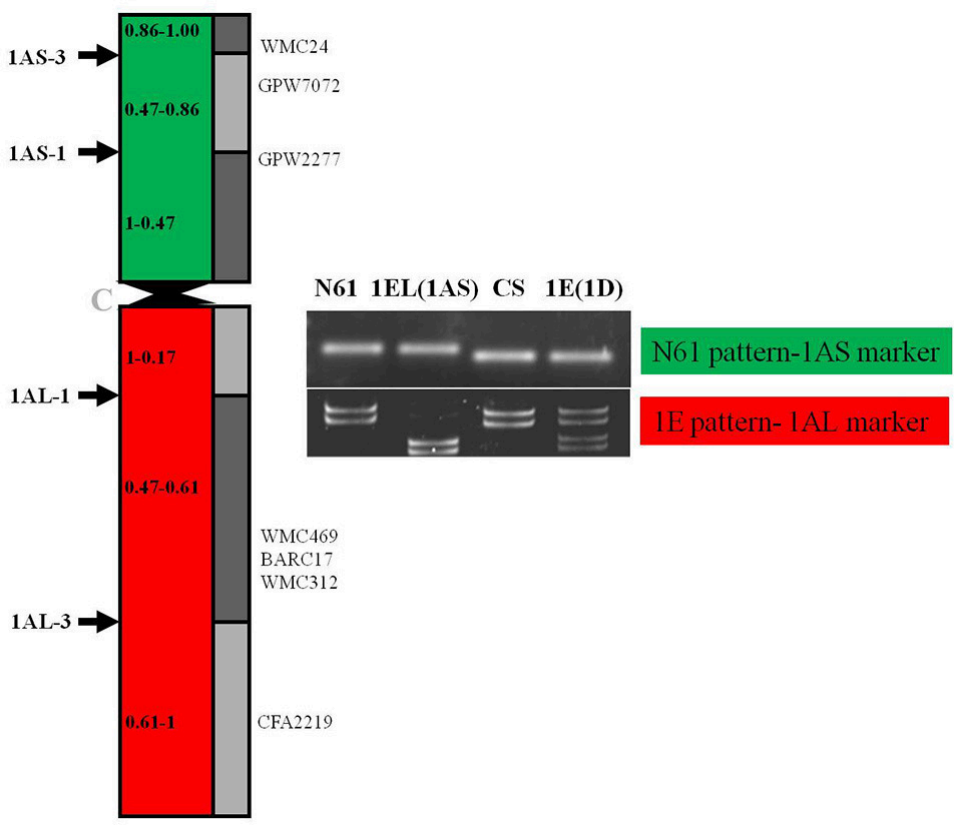
Schematic representation of translocated chromosome 1EL(1AS) in the DTL-1EL(1AS) to indicate number and position of SSR markers; the 1AS region showed N61 pattern, while long arm specific markers showed *Th. elongatum* specific pattern.

The BC_4_F_3_ derivatives of the confirmed translocation line in the background of Japanese cultivar Norin 61 were screened for background recovery. Total 536 SSR markers were utilized for this purpose. Out of 536, total 119 markers were found to be polymorphic. Background screening of the lines revealed that the translocation lines had mostly Norin 61 background (Figure 5; Supplementary Table 1). For chromosomes 1B, 1D, 2A, 2B, 2D all the polymorphic SSRs showed N61. CS pattern was observed in a very less number of cases; 3A, 3B, and 7B in one SSR each. Rest for all the chromosomes, N61 pattern was observed indicating good recovery of parent background. For chromosome 1A, total 41 SSR markers were used out of which 19 were found to be polymorphic. We observed N61 pattern using short arm region specific primers and *Th. elongatum* banding pattern using long arm specific primers (Figure 6). The markers which showed *Th. elongatum* pattern were WMC469, BARC17, WMC312, and CFA2219.

### Morphological characters

**Figure 7:**
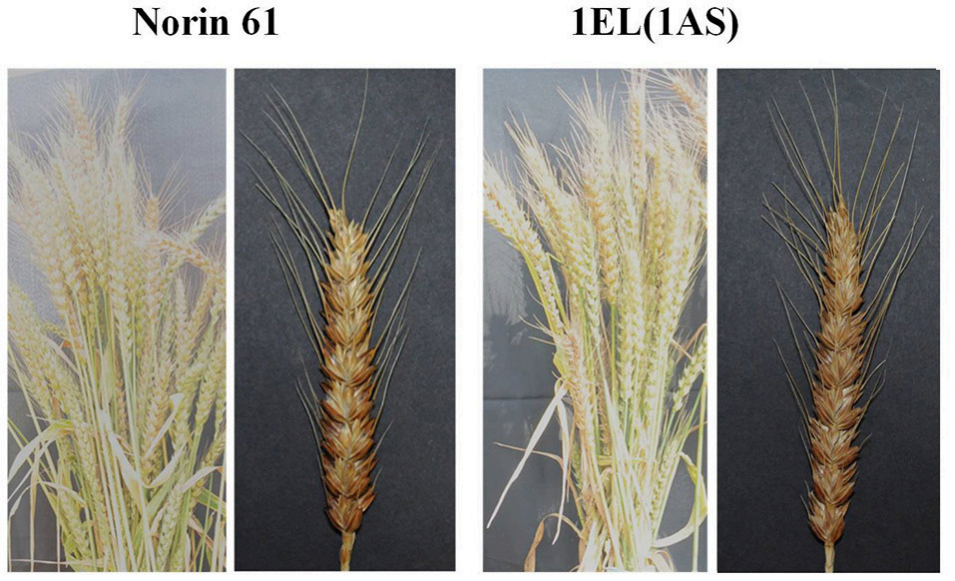
Morphological similarity of DTL-1EL(1AS) with Norin 61.

Morphologically the translocation line 1EL(1AS) plants were similar to parent N61 (Figure 7). We measured plant height, spike length, awn length, number of spikelets, flag leaf length, and width for N61 and DTL-1EL(1AS; Table 2). Spike length of DTL-1EL(1AS) was higher as compared to N61 but no differences were observed in a number of spikelets. There were no significant differences observed in plant height, awn length, flag leaf length, and width.

**Table 2:** Morphological data of translocation line (BC_4_F_6_-NIL) in comparison to recipient cultivar N61.

**Parameters**	**Norin61**	**1EL(1AS)**
Plant height (cm)	102.6 ± 5.42^a^	104.67 ± 4.58^a^
Spike length (cm)	8.5 ± 0.65^a^	9.3 ± 0.96^b^
Awn length (cm)	4.11 ± 0.78^a^	3.89 ± 0.89^a^
Spikelets/spike	18 ± 2^a^	18 ± 2^a^
Flag leaf length (cm)	17.79 ± 3.73^a^	18.92 ± 2.23^a^
Flag leaf width (cm)	1.49 ± 0.28^a^	1.51 ± 0.18^a^

*Translocation line was morphologically similar to N61. Values followed by same letter in column are not significantly different at p > 0.05*.

### Scanning electron microscopy (SEM) of seeds

**Figure 8:**
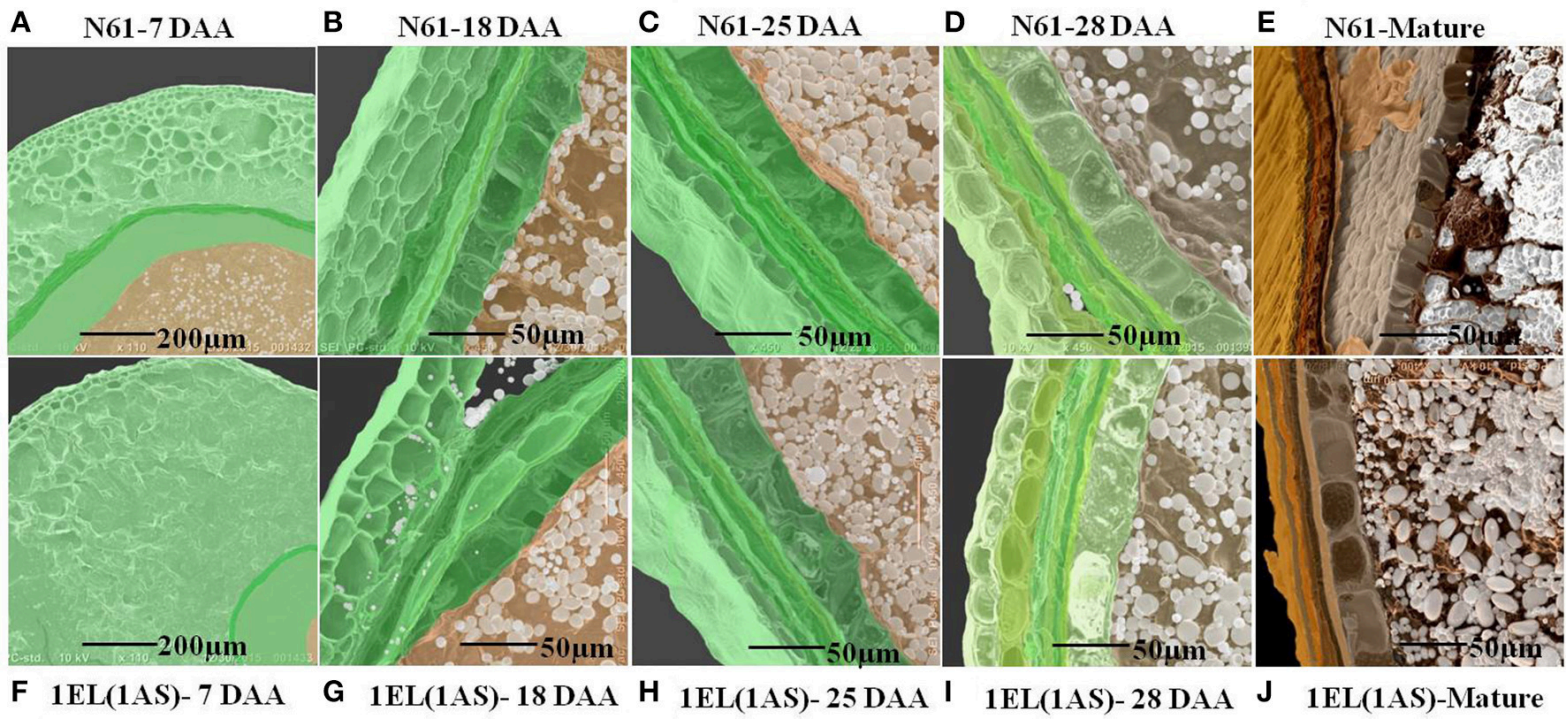
Scanning electron microscopy of different seed development stages starting from 7 days after anthesis (7 DAA) to 28 DAA of N61 and DTL-1EL(1AS). SEM images indicated that the translocation line showed slow development at 7 DAA as compared to N61. But for the later development stages there was no significant difference observed. **(A)** N61(7 DAA), **(B)** N61(18 DAA), **(C)** N61(25 DAA), **(D)** N61(28 DAA), **(E)** N61(mature seeds), **(F)** 1EL(1AS)(7 DAA), **(G)** 1EL(1AS) (18 DAA), **(H)** 1EL(1AS)(25 DAA), **(I)** 1EL(1AS) (28 DAA), **(J)** 1EL(1AS) (mature seeds).

The scanning electron microscopy was performed on four different developmental stages (7 days after anthesis {DAA}, 18 DAA, 25 DAA, and 28 DAA) and mature seeds {Figures 8A–E for N61, Figures 8F–J for DTL-1EL(1AS)}. The results showed that the translocation line showed slow development at 7 DAA as compared to N61 (Figures 8A,F). But for the lateral developmental stages, there were no significant differences observed. Seed development of translocation line and N61 were similar at 18 DAA (Figures 8B,G), 25 DAA (Figures 8C,H), 28 DAA (Figures 8D,I). The SEM images of mature seeds, indicated similar packing of starch granules in translocation line and N61 (Figures 8E,J).

### Processing quality parameters

**Figure 9:**
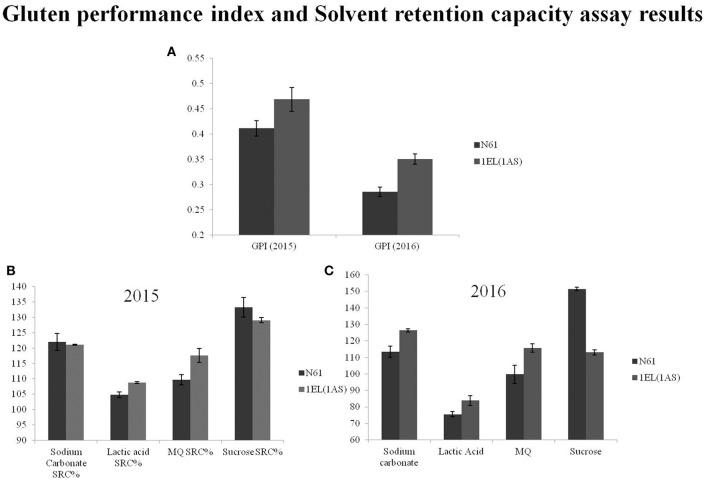
SRC data and GPI-values for Norin 61 and DTL-1EL(1AS); **(A)** Gluten performance index (GPI)-values for the year 2015 and 2016; **(B)** SRC-values for the year 2015; **(C)** SRC-values for the year 2016. SRC-values and GPI indicated improved dough strength in translocation line.

We did not see any significant differences in total protein content between N61 and DTL-1EL(1AS) over a period of 3 years (2014–2016; Table 3). Similarly, dry gluten and wet gluten content remained similar in both N61 and DTL-1EL(1AS). However, variations were observed in values of different years, which might be contributing to different environmental conditions at different times. The values of grain hardness also remained similar for both N61 and DTL-1EL(1AS). The thousand kernel weight (TKW) for translocation line was less as compared to N61. The value of test weight remained same in both N61 and DTL-1EL(1AS). There was a significant increase in SDS sedimentation values for DTL-1EL (1AS) as compared to parent N61. Dough extensibility tests showed an increase in peak positive force, stretching distance and area to positive peak in the case of DTL-1EL(1AS) as compared to parent N61 (Table 4). The results were consistent for 2 years (2015, 2016). These results indicated improvement in dough extensibility properties of translocation line as compared to parent N61. Mixograph analysis also showed increases in midline peak time, midline peak value and midline peak integral in case of DTL-1EL(1AS) as compared to N61 (Table 4). There were no significant differences observed in values of midline peak width. Thus, overall mixing parameters for DTL-1EL (1AS) were shown to improve indicating improvement in dough strength. Solvent retention capacity test was carried out to confirm dough strength (Figure 9). In this test four different solutions of lactic acid, sucrose, water, and sodium carbonate were utilized for providing information on physical and chemical aspects of wheat samples. No difference was observed in the sodium carbonate retention capacity for translocation lines and parent N61 in the year 2015. The value increased in the year 2016. There was a significant increase in lactic acid SRC in DTL-1EL(1AS) in both years. Reverse trend was observed in case of sucrose SRC where the values decreased in DTL-1EL(1AS) in both years. There was an increase in MQ-SRC values of the translocation line in both years. The SRC values were used to calculate gluten performance index (GPI) of the flour. The results of SRC showed an increase in GPI of translocation line when compared to parent N61. Dough strength of translocation line was better than N61. Similar results were observed for both the studied years.

**Table 3:** Quality parameters across three years of translocation line in comparison to recipient cultivar.

**Cultivar/Line**	**Protein content (%)**	**Dry gluten (%)**	**Wet gluten (%)**	**Thousand kernel weight (g)**	**Test weight (g)**	**Grain hardness index**	**SDS sedimentation value**
N61 (2014)	10.53 ± 0.48^a^	8.07 ± 0.53^a^	23.85 ± 2.45^a^	35.38 ± 0.63^a^	74.3 ± 0.36^a^	31	2.78 ± 0.04^a^
1EL(1AS) (2014)	10.65 ± 0.44^a^	8.4 ± 0.72^a^	22.67 ± 2.54^a^	32.15 ± 0.49^b^	74.6 ± 0.71^a^	30	3.64 ± 0.16^b^
N61 (2015)	8.6 ± 1.2^a^	6.1 ± 1.1^a^	17.9 ± 1.6^a^	33.63 ± 0.76^a^	75.8 ± 0.9^a^	33.93	2.37 ± 0.058^a^
1EL(1AS) (2015)	8 ± 0.3^a^	5.9 ± 0.3^a^	17.2 ± 0.6^a^	30.47 ± 2.33^b^	74.9 ± 0.5^a^	35.15	3 ± 0.1^b^
N61 (2016)	9.33 ± 0.68^a^	5.83 ± 0.45^a^	18.53 ± 1.63^a^	34.67 ± 0.51^a^	78.33 ± 0.74^a^	27	2.34 ± 0.10^a^
1EL(1AS) (2016)	8.87 ± 0.61^a^	5.03 ± 0.67^a^	17.63 ± 1.40^a^	28 ± 3.28^b^	77.077 ± 0.68^a^	33	2.81 ± 0.18^b^

*Multi-environment data indicated that improvement in dough strength in the translocation line was due to genetic factors. Values followed by same letter in column are not significantly different at p > 0.05*.

**Table 4:** Dough extensibility and mixing properties of translocation line in comparison to recipient cultivar.

**Line**	**Peak positive force (g)**	**Area to positive peak (g.s)**	**Stretching distance (mm)**	**Midline peak time (min)**	**Midline peak value (%)**	**Midline peak width (%)**	Midline peak integral (%tq^*^min)
N61 (2015)	26.90 ± 0.48^a^	59.67 ± 7.93^a^	11.06 ± 0.80^a^	3.03 ± 0.27^a^	48.84 ± 2.71^a^	14.20 ± 2.31^a^	136.45 ± 7.83^a^
1EL(1AS) (2015)	30.59 ± 1.96^b^	90.13 ± 10.02^b^	13.25 ± 0.97^b^	4.99 ± 0.64^b^	43.96 ± 2.78^b^	12.67 ± 2.19^a^	198.64 ± 5.33^b^
N61 (2016)	28.5 ± 1.45^a^	60.16 ± 5.06^a^	10.25 ± 0.63^a^	3.53 ± 0.13^a^	54.84 ± 1.71^a^	16.20 ± 2.31^a^	156.70 ± 10.32^a^
1EL(1AS) (2016)	36.02 ± 3.96^b^	93.18 ± 16.03^b^	12.87 ± 1.19^b^	5.57 ± 0.54^b^	48.96 ± 2.78^b^	14.67 ± 2.19^a^	233.94 ± 7.84^b^

*Multi-environment data indicated that improvement in mixing properties and extensibility in the translocation line was due to genetic factors. Values followed by same letter in column are not significantly different at p > 0.05*.

## Discussion

Wheat is one of the most important staple cereal crops consumed by billions across the world. Wide introgression in wheat to broaden its genetic base has resulted in significant improvement in productivity, disease resistance, and agricultural economy (Rabinovich, 1998). The conventional approach for development of alien introgression is through generation of amphiploids by crossing wheat directly with wild species and then screening and selecting subsequent generations for individual alien chromosome additions or substitutions (Qi et al., 2007; Wang et al., 2013). Whole chromosome introgression in form of additions or substitutions is usually associated with linkage drag causing unwanted agronomic traits (Zamir, 2001). Better alternative is to transfer small chromosome segment by means of translocations. Many chromosome engineering approaches have been used to transfer small segments of alien chromosomes without linkage drag (Mukai et al., 1993; Lukaszewski, 1997; Qi et al., 2007). Radiation treatment and gametocidal genes have been utilized for development of wheat-rye (Ahmad et al., 2000; Masoudi-Nejiad et al., 2002), - *Aegilops umbellulata* (Sears, 1956), and wheat-*Leymus racemosus* translocations (Chen et al., 2005). Both ionizing treatment and gametocidal genes induce random chromosome breakage and thus random translocations with often undesirable agronomic traits (Qi et al., 2007). The availability of nulli-terta genetic stocks (Sears, 1965) of hexaploid wheat makes it possible to create chromosome specific translocation using addition and substitution lines. Marais et al. (2009) have used addition line of *Aegilops neglecta* and crossed it with CS followed by repeated backcrossing and screening for development of translocations. Similar methodology was adopted by Kuraparthy et al. (2009) where they used substitution line 5Mg(5D) and crossed with CS(*Ph*^*I*^) stocks for development of translocations. We utilized N1AT1D genetic stock along with DSL-1E(1D) as a targeted approach to replace chromosome 1AL with chromosome 1EL. The cross between N1AT1D and DSL-1E(1D) produced F1 generation which was double monosomic for both chromosomes 1A and 1E. Double monosomics were supposed to produce rare translocation in the next generation. We also selected for Disomic substitution DSL-1E(1A) in the next generation that was expected to produce double monosomics in next cross. The chances of homoeologous pairing between 1A and 1E were thus increased by selecting initial genetic material.

The chromosome specific translocation line of wheat generated in the current manuscript [1EL(1AS)] thus is a unique example of targeted wide introgression achieved by carefully selecting the breeding material and the screening technologies. To the best of our knowledge this is the first, unique approach targeted to improve wheat processing quality. Previously, Wang et al. (2013) and Garg et al. (2014) developed *Aegilops longissima* specific disomic substitution lines 1Sl(1B) and 1Sl(1A), which were reported to possess superior bread making quality. Similarly disomic addition lines of *Aegilops searsii* in CS background have been reported to possess improved dough strength (Garg et al., 2009b). But linkage drag and genetic instability makes use of addition/substitution lines as direct cultivar impractical. The basic concept was the utilization of *Th. elongatum* potential while retaining the existing wheat qualities. We targeted chromosome 1A as its HMW-GS have least contribution toward wheat dough strength. We wanted to retain both 1D and 1B specific HMW-GS due to their significant positive effect (Rogers et al., 1990; Kumlay et al., 2003; Garg et al., 2014). This involved our previous work on understanding the contribution of individual wheat and wild relative chromosomes by screening a large number of addition lines (Garg et al., 2009b, 2014).

Creation of translocation line is costly, long, and tedious process, involving screening of a large number of entries with very low success frequency (Ko et al., 2002). We have reduced the time, cost, and efforts involved by combining protein marker screening with GISH/FISH. GISH is the common technique to identify translocation that is costly, time consuming, and requires significant trained manpower hours. We first screened our carefully selected breeding material with chromosome specific protein markers from the endosperm half and raised the selected seedling from embryo part, collected first two roots for mitotic metaphase analysis and used rest of the seedling to raise plant. The selected seeds were screened by GISH. With this strategy, we were able to increase screening efficiency at the second stage to as high as 33.3%. Interestingly, we have developed this translocation from normal cross rather than homoeologous pairing suppressor mutant (*Ph*^*I*^) cross. In the first screening cycle, we could not succeed in our efforts, so we involved *Ph*^*I*^ line in our crosses. Wheat is allohexaploid with three different genomes and pairing takes place between homologous pairs of chromosomes that makes wide introgression a difficult task. Wheat researchers have tried to circumvent this challenge by deleting the chromosome part that does not allow homoeologous recombination. Using this approach several translocation lines have been generated (Sears, 1976; Rey et al., 2015). Rather, our breeding material with *Ph*^*I*^ was poor in growth, seed setting and we could not use this material for screening. We expect that multiple deletion/translocation events might have had a detrimental effect on plant growth.

We have not only generated translocation line, but also recovered the background in one of the leading cultivars: N61 by several cycles of backcrossing/selfing (BC_4_F_6_) and thus developed almost near isogenic line (NIL). We have characterized it and carried out two year quality analysis. This line showed improved dough strength, mixing properties, and extensibility compared to N61 recipient parent. Our multi environment data indicated that improvement in the translocation line was due to genetic factors. This is a unique material that can be utilized in many breeding programs targeting grain processing quality. This material has been transferred to researchers and breeders for its utilization.

## Author contributions

Conceived and designed the experiments: AmK, MG, and HT. Performed the experiments: AmK, MG, NK, SM, and QD. Analyzed the data: AmK, MG, and AsK. Contributed reagents/materials/analysis tools: VC, RG, and SS. Wrote the paper: AmK and MG.

### Conflict of interest statement

The authors declare that the research was conducted in the absence of any commercial or financial relationships that could be construed as a potential conflict of interest.
