# Supplementary material for: Rapid Development and Characterization of Chromosome Specific Translocation Line of Thinopyrum elongatum with Improved Dough Strength
Source: Front Plant Sci. 2017 Sep 14;8:1593. doi: 10.3389/fpls.2017.01593 (PMC5604074; doi:10.3389/fpls.2017.01593)
Supplement: Supplementary file 1 [file Presentation1.PPTX]

## Slide 1
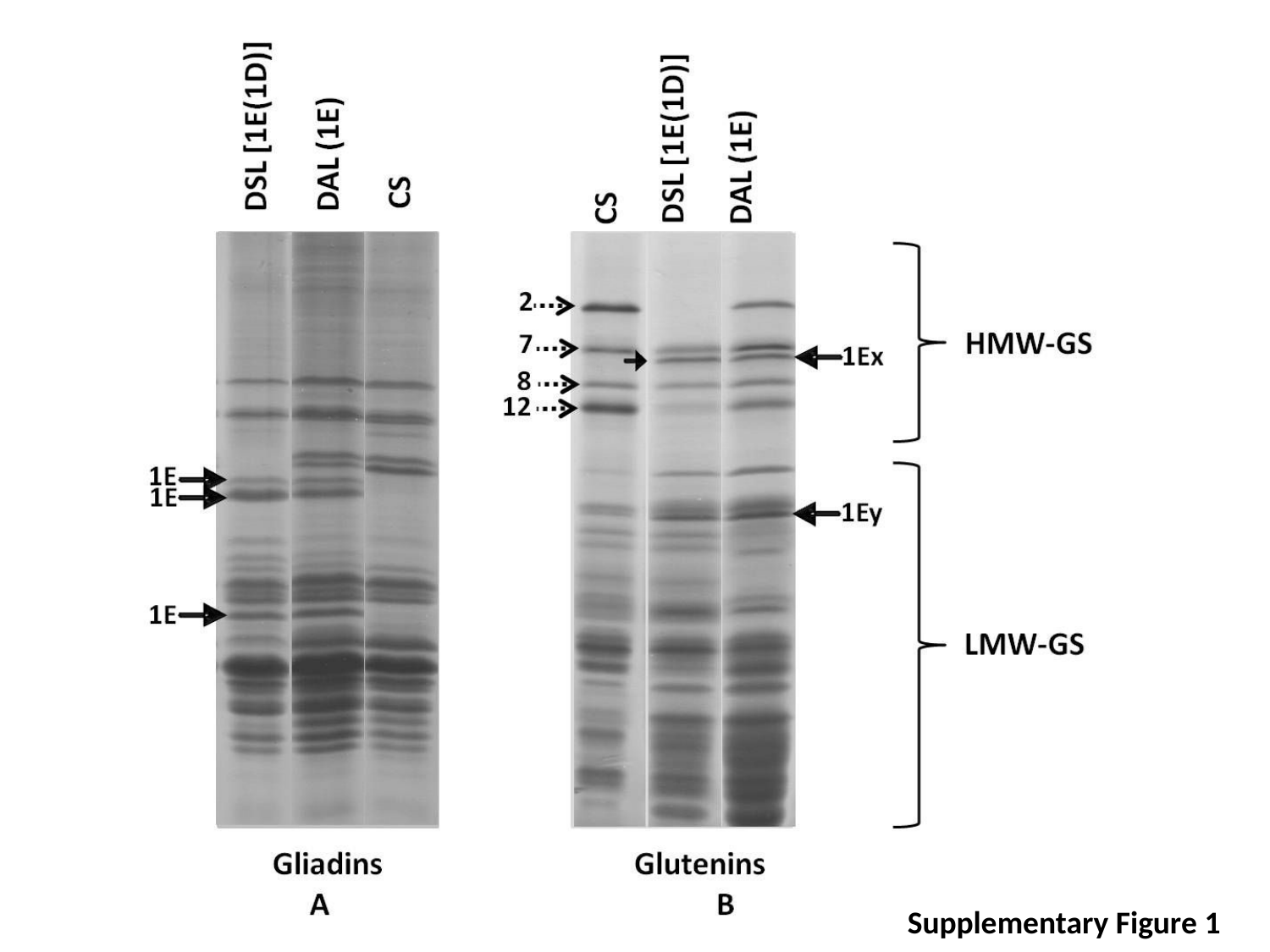

Supplementary Figure 1

## Slide 2
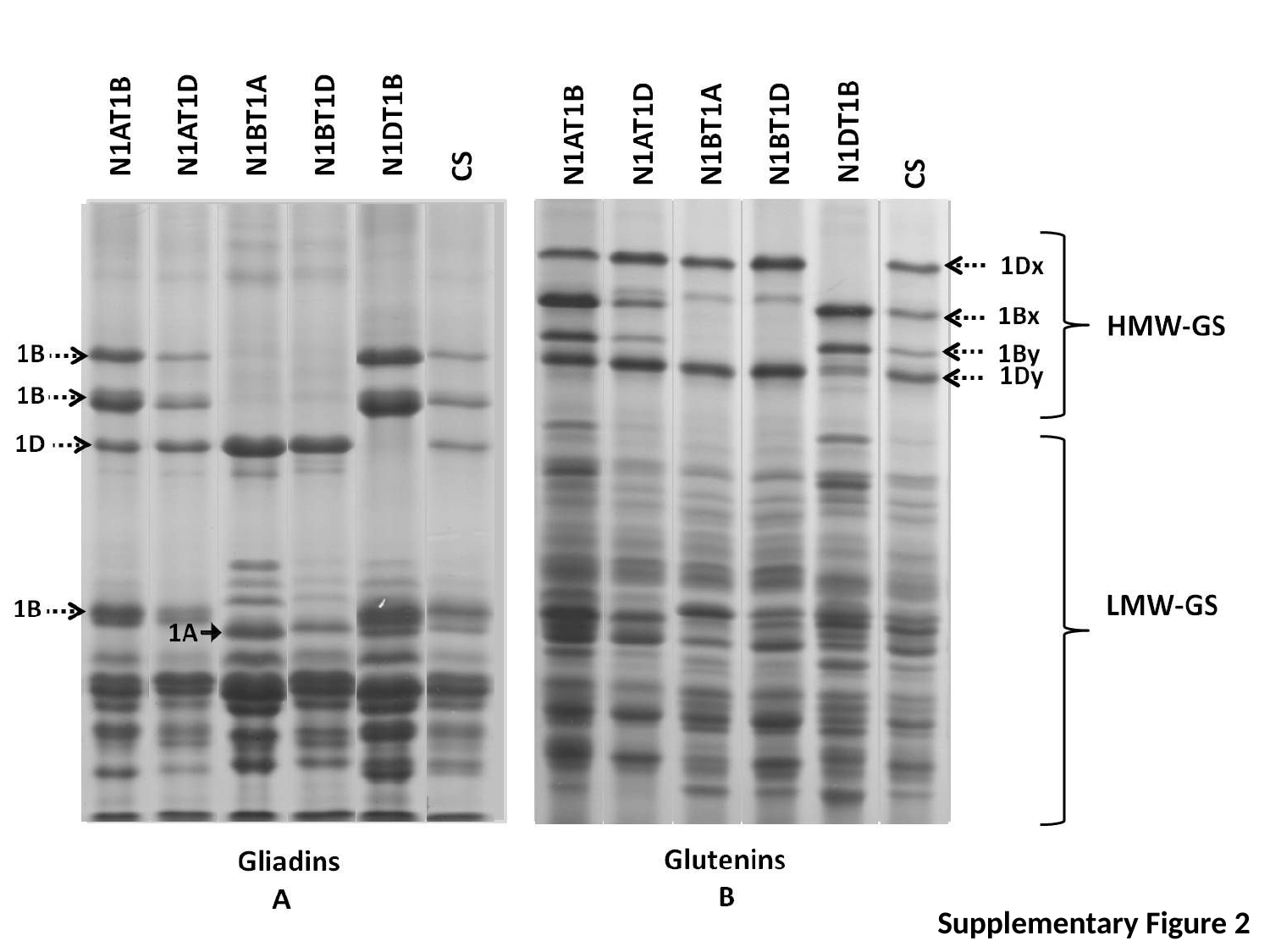

Supplementary Figure 2

## Slide 3
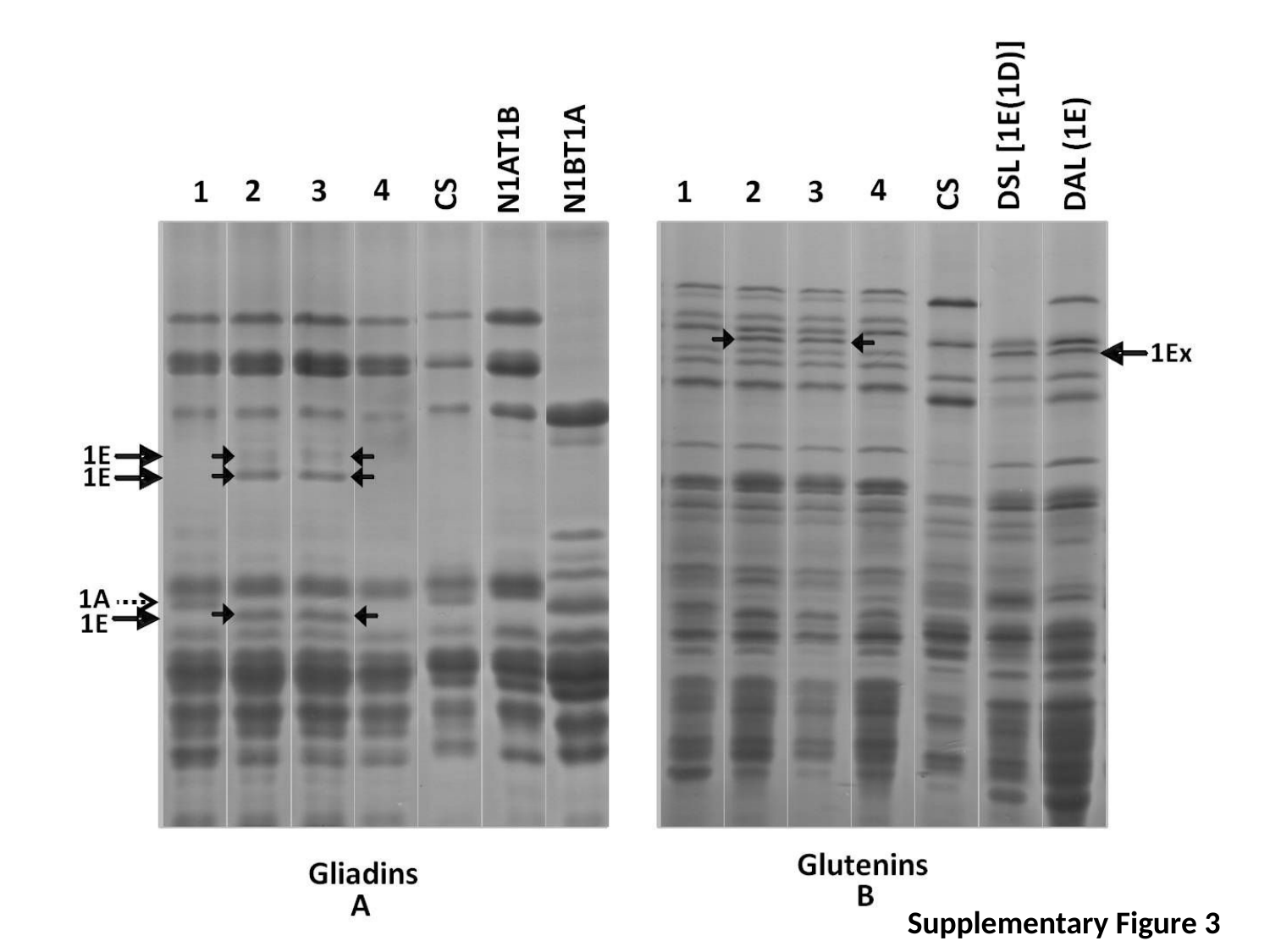

Supplementary Figure 3

## Slide 4
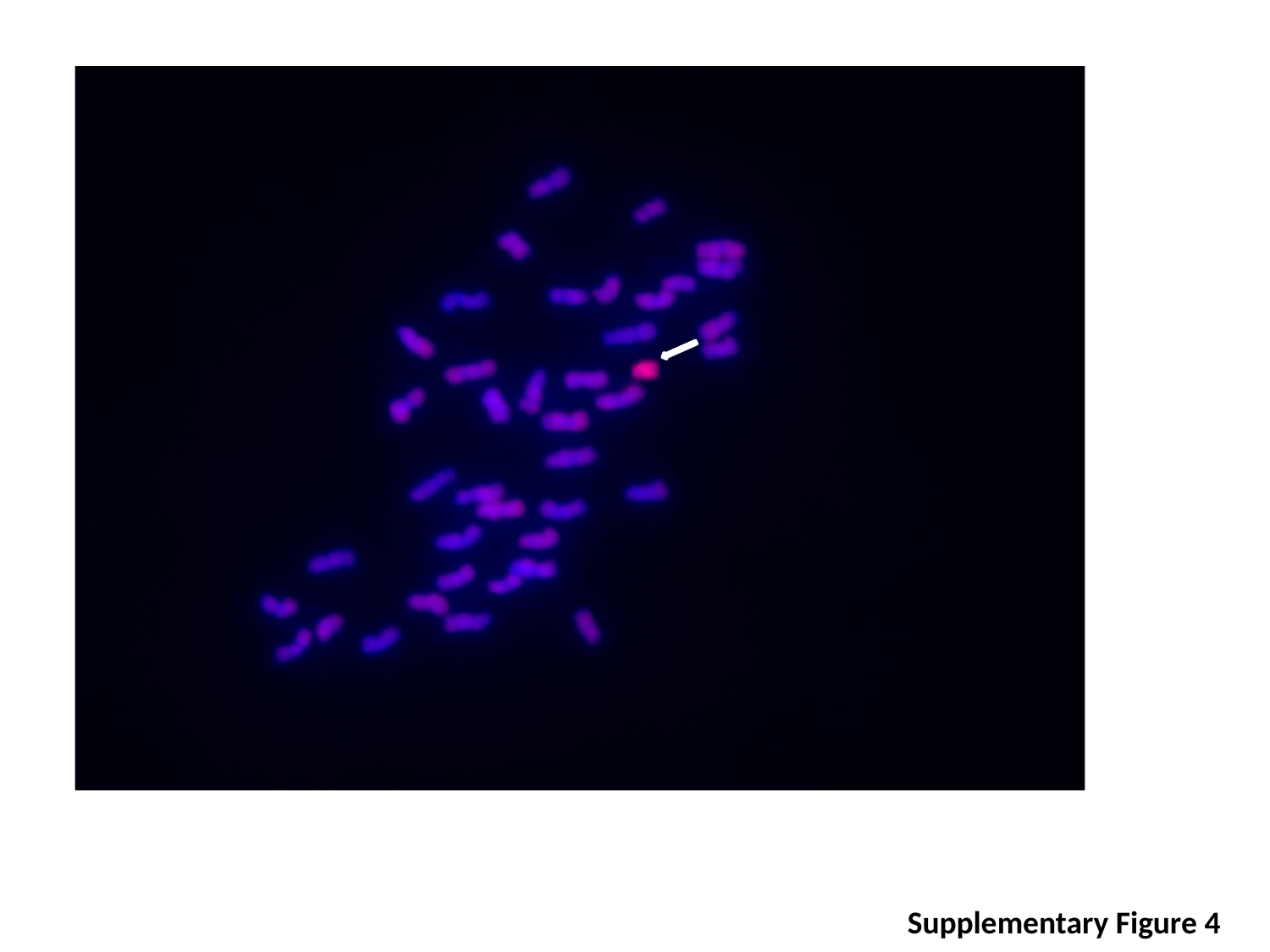

Supplementary Figure 4

## Slide 5
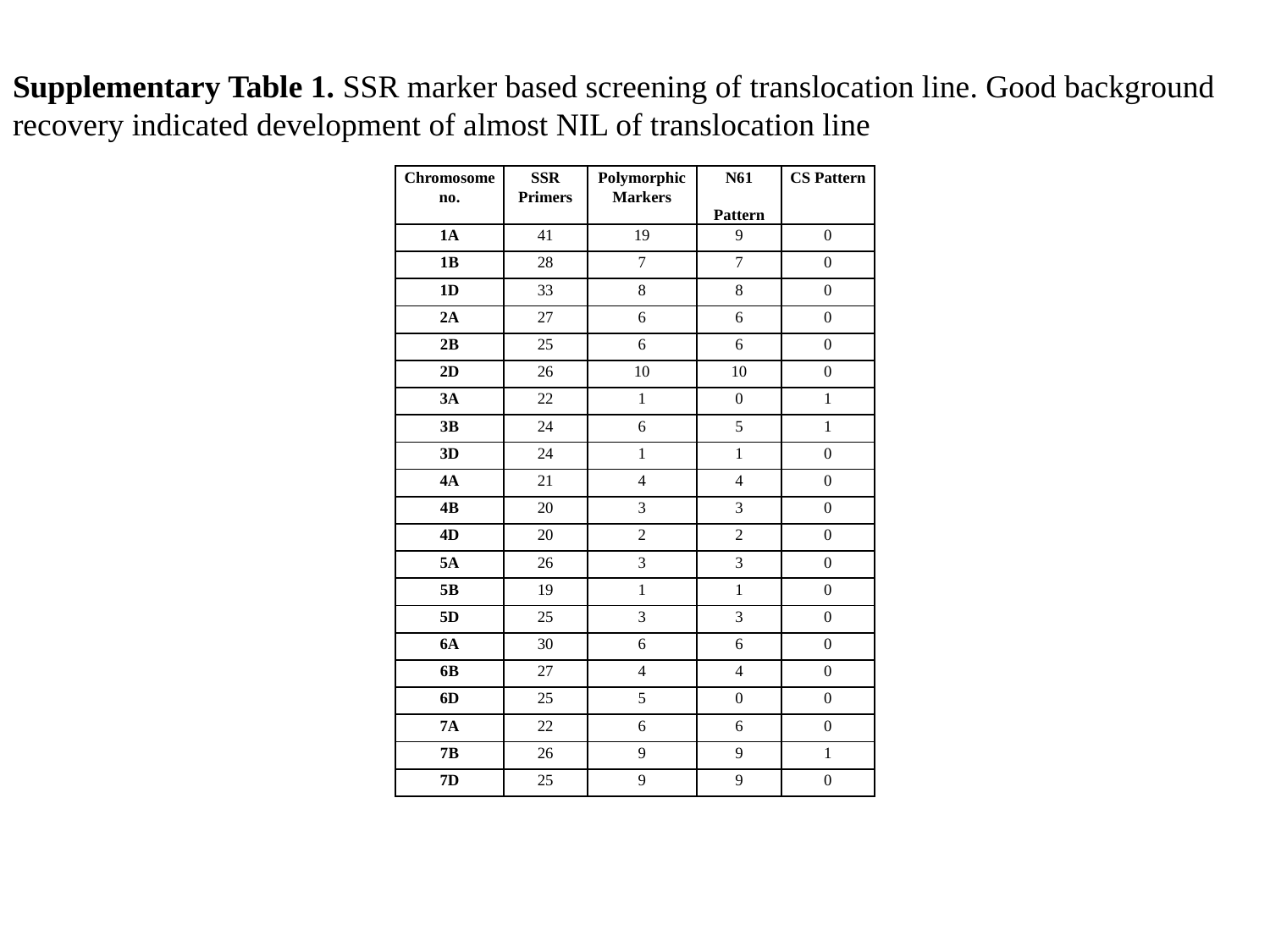

Supplementary Table 1. SSR marker based screening of translocation line. Good background recovery indicated development of almost NIL of translocation line
| Chromosome no. | SSR Primers | Polymorphic Markers | N61 Pattern | CS Pattern |
| --- | --- | --- | --- | --- |
| 1A | 41 | 19 | 9 | 0 |
| 1B | 28 | 7 | 7 | 0 |
| 1D | 33 | 8 | 8 | 0 |
| 2A | 27 | 6 | 6 | 0 |
| 2B | 25 | 6 | 6 | 0 |
| 2D | 26 | 10 | 10 | 0 |
| 3A | 22 | 1 | 0 | 1 |
| 3B | 24 | 6 | 5 | 1 |
| 3D | 24 | 1 | 1 | 0 |
| 4A | 21 | 4 | 4 | 0 |
| 4B | 20 | 3 | 3 | 0 |
| 4D | 20 | 2 | 2 | 0 |
| 5A | 26 | 3 | 3 | 0 |
| 5B | 19 | 1 | 1 | 0 |
| 5D | 25 | 3 | 3 | 0 |
| 6A | 30 | 6 | 6 | 0 |
| 6B | 27 | 4 | 4 | 0 |
| 6D | 25 | 5 | 0 | 0 |
| 7A | 22 | 6 | 6 | 0 |
| 7B | 26 | 9 | 9 | 1 |
| 7D | 25 | 9 | 9 | 0 |
